# Supplementary material for: Prognostic and chemotherapeutic implications of a novel four-gene pyroptosis model in head and neck squamous cell carcinoma
Source: PeerJ. 2024 May 13;12:e17296. doi: 10.7717/peerj.17296 (PMC11097961; doi:10.7717/peerj.17296)
Supplement: Supplemental Information 1 [file peerj-12-17296-s001.docx]

**Figure S1 The prognostic significance of the PRGscore was verified in the GEO cohort**

**
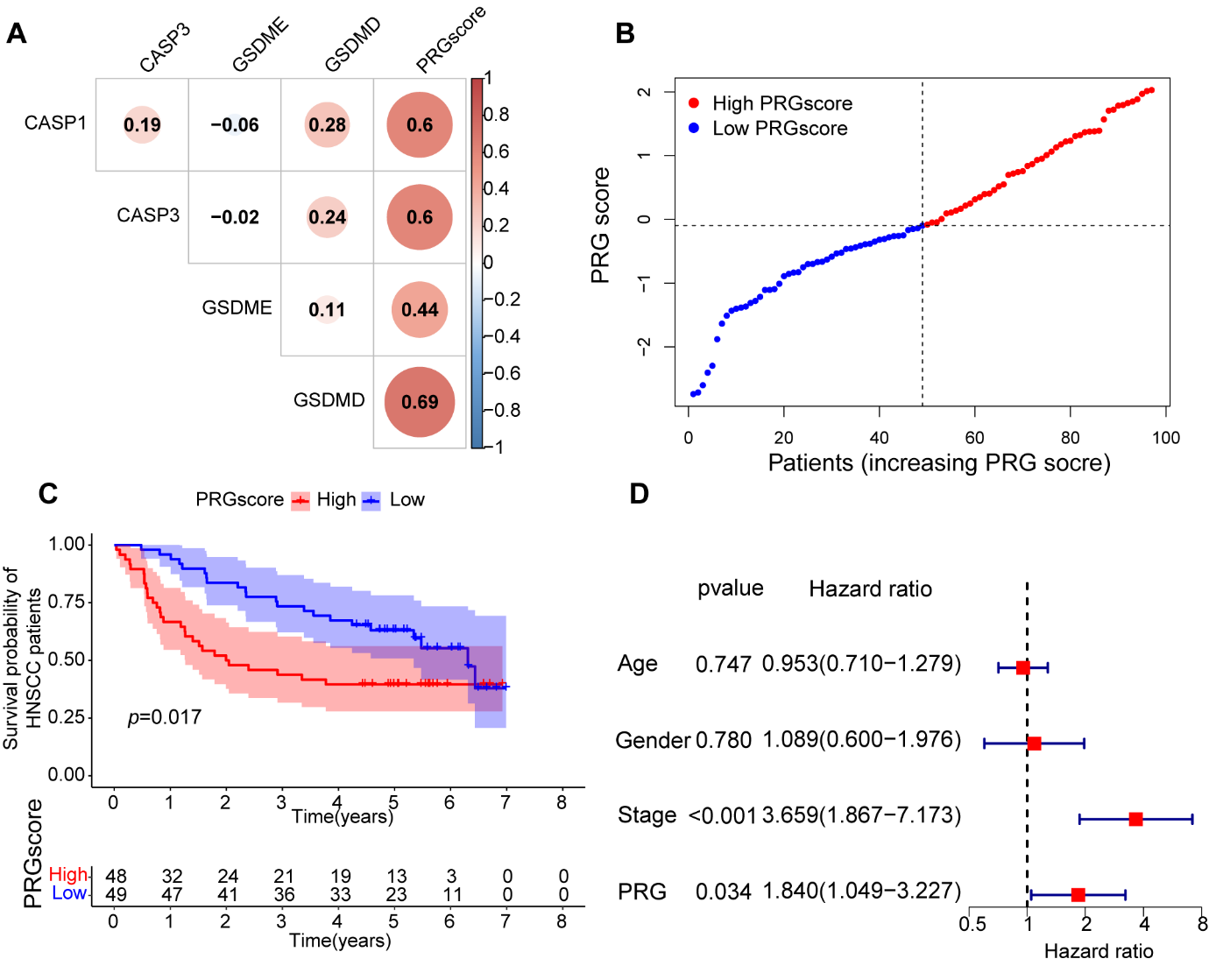
**

**Fig. S1.** **The prognostic significance of the PRGscore was verified in the GEO cohort**. (A) Pearson correlation analysis between 4 pyroptosis-related genes and PRGscore. (B) PRGscore of HNSCC samples premised on the cutpoint (the left of the dotted line signifies the low PRGscore group while the right side signifies the high PRGscore group). (C) Kaplan-Meier survival analysis of low and high PRGscore subgroups; the dashed lines represent the 95% confidence interval. (D) Multivariate Cox regression analysis of the PRGscore and clinicopathological parameters in HNSCC samples. Abbreviations: GEO, The Gene Expression Omnibus; CASP1, Caspase-1; CASP3, Caspase-3; GSDMD, Gasdermin D; GSDME, Gasdermin E; PRGscore, Pyroptosis-Related Gene Score; HNSCC, head and neck squamous cell carcinoma.

**Figure S2 Relative expression levels of 4 genes in 14 HNSCC cell lines.**


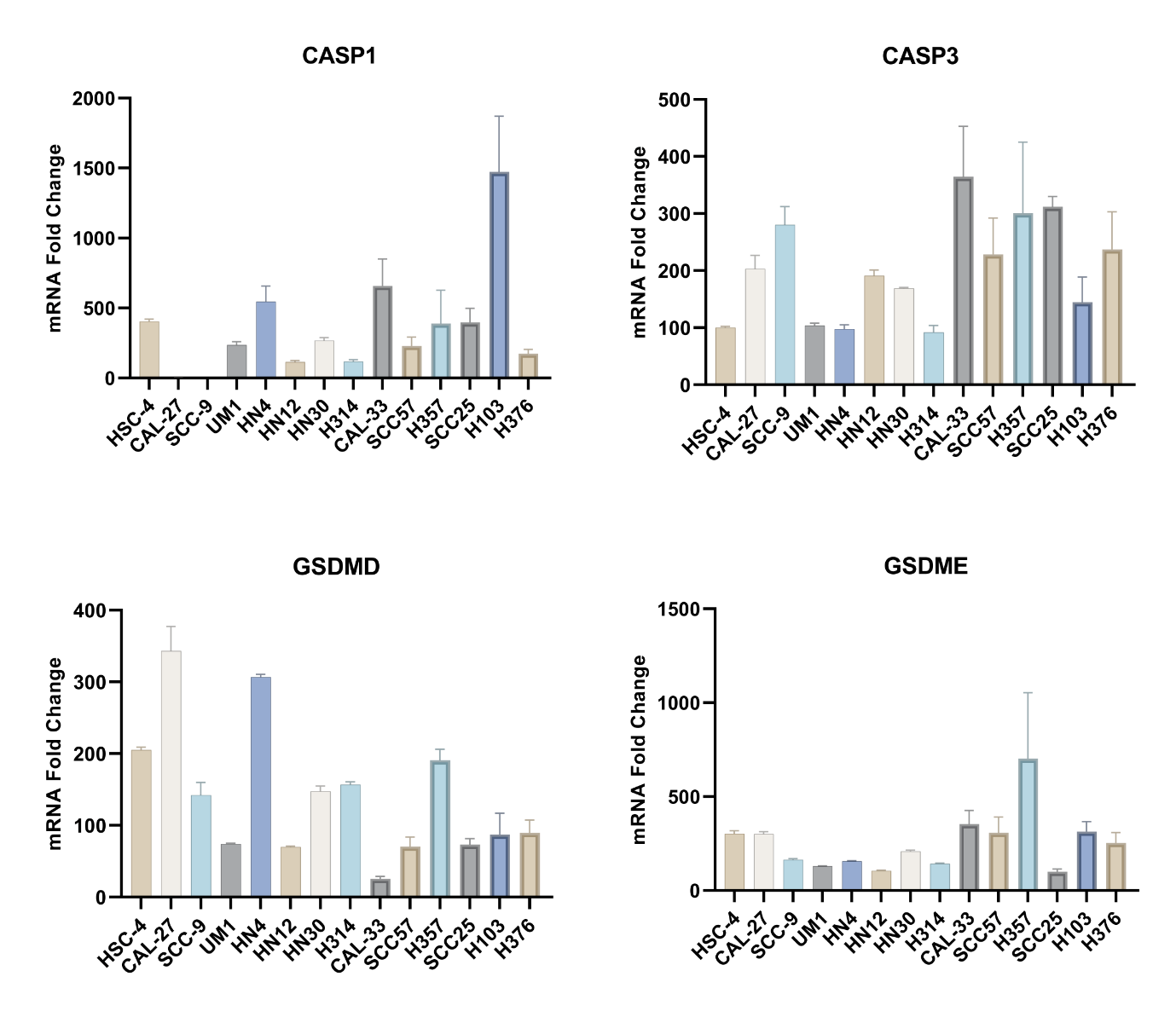
**Fig. S2. Relative expression levels of 4 genes in 14 HNSCC cell lines.** Abbreviations: CASP1, Caspase-1; CASP3, Caspase-3; GSDMD, Gasdermin D; GSDME, Gasdermin E; HNSCC, Head and neck squamous cell carcinoma.

**Figure S3 Evaluation of the correlation between PRGscore and potential response to immunotherapy for HNSCC patients
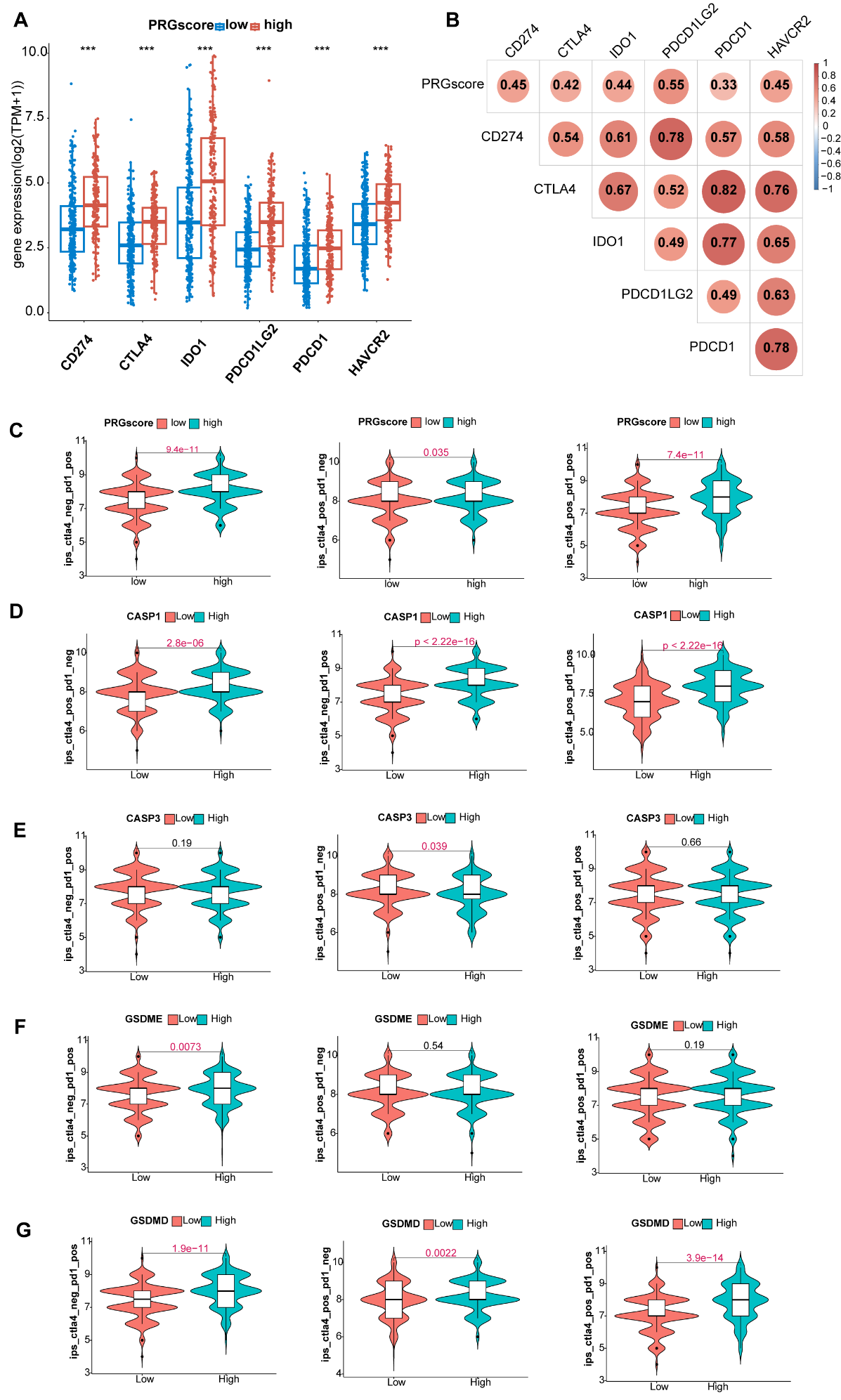
**

**Fig. S3. The correlation between PRGscore and immunotherapy efficacy for HNSCC patients.** (A) Differential expression analysis of immune checkpoint genes (CD274, CTLA4, PDCD1, IDO1, PDCD1LG2, and HAVCR2) between high and low PRGscore groups. (B) Pearson correlation analysis between PRGscore and several key immune checkpoints’ expression level. (C) The association between the PRGcore or pyroptosis-related genes and the immunopheno score of anti-PD1 monotherapy, anti-CTLA4 monotherapy and the combination of anti-PD1 and anti-CTLA4 immunotherapy. Abbreviations: PRGscore, Pyroptosis-Related Gene Score; HNSCC, head and neck squamous cell carcinoma; CD274, Programmed Cell Death 1 Ligand 1; CTLA4, Cytotoxic T-Lymphocyte-Associated Protein 4; PDCD1, Programmed Cell Death 1((PD-1)); IDO1, Indoleamine 2,3-Dioxygenase 1; PDCD1LG2, Programmed Cell Death 1 Ligand 2; HAVCR2, Hepatitis A Virus Cellular Receptor 2.

**Figure S4 Evaluation of the correlation between PRGscore and potential response to immunotherapy for HNSCC patients**


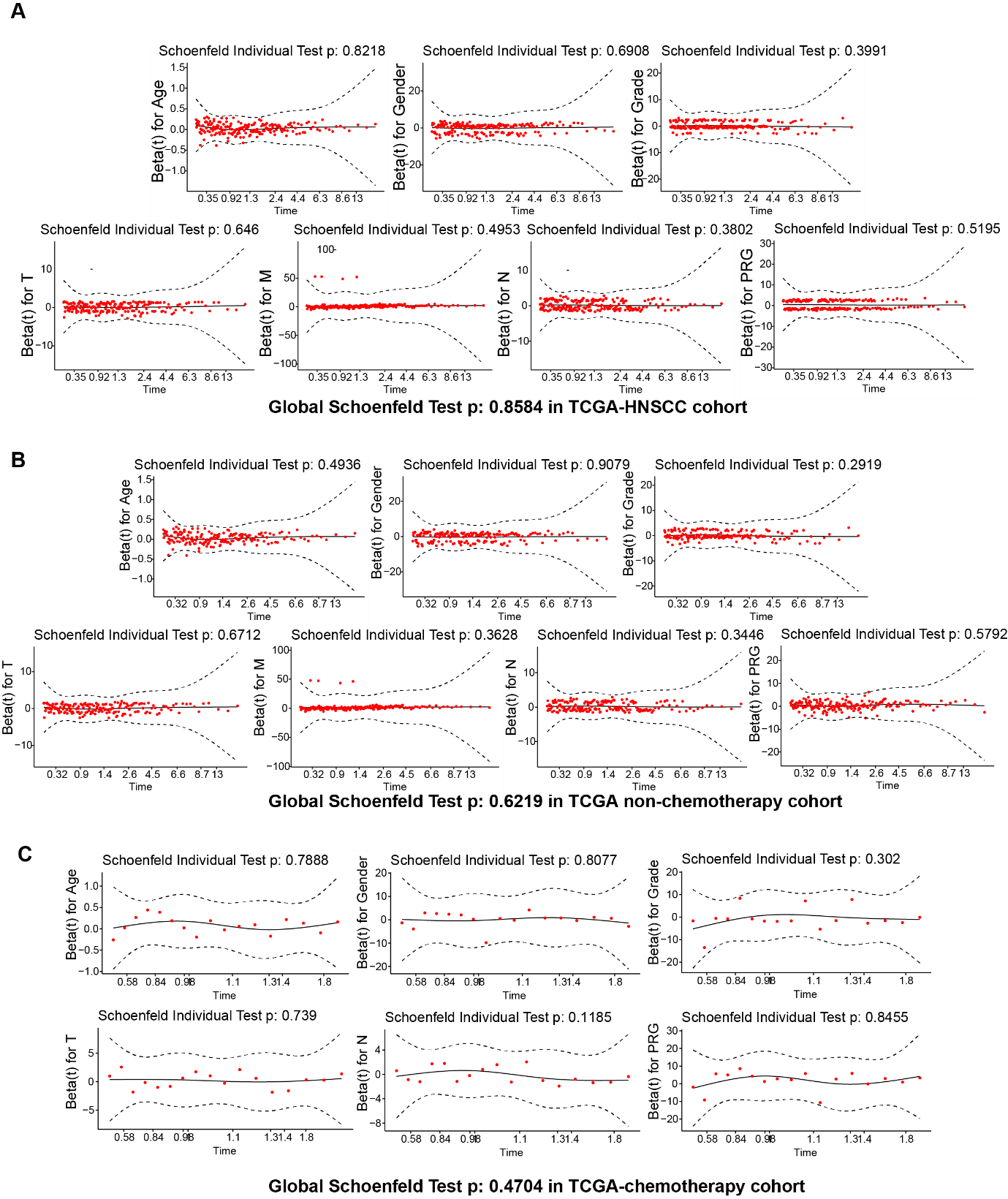
**Fig. S4. Graphs of Schoenfeld residuals.** (A) Schoenfeld residuals test result in TCGA-HNSCC cohort. (B) Schoenfeld residuals test result in TCGA-HNSCC nonchemotherapy cohort. (C) Schoenfeld residuals test result in TCGA- HNSCC chemotherapy cohort. The solid line in the figure is the smooth spline fit of the curve, and the broken lines represent a ± 2-standard-error band around the fit.**Figure S5 Construction of the pyroptosis-related prognostic signature (TCGA cohort) based on 2 genes**

**
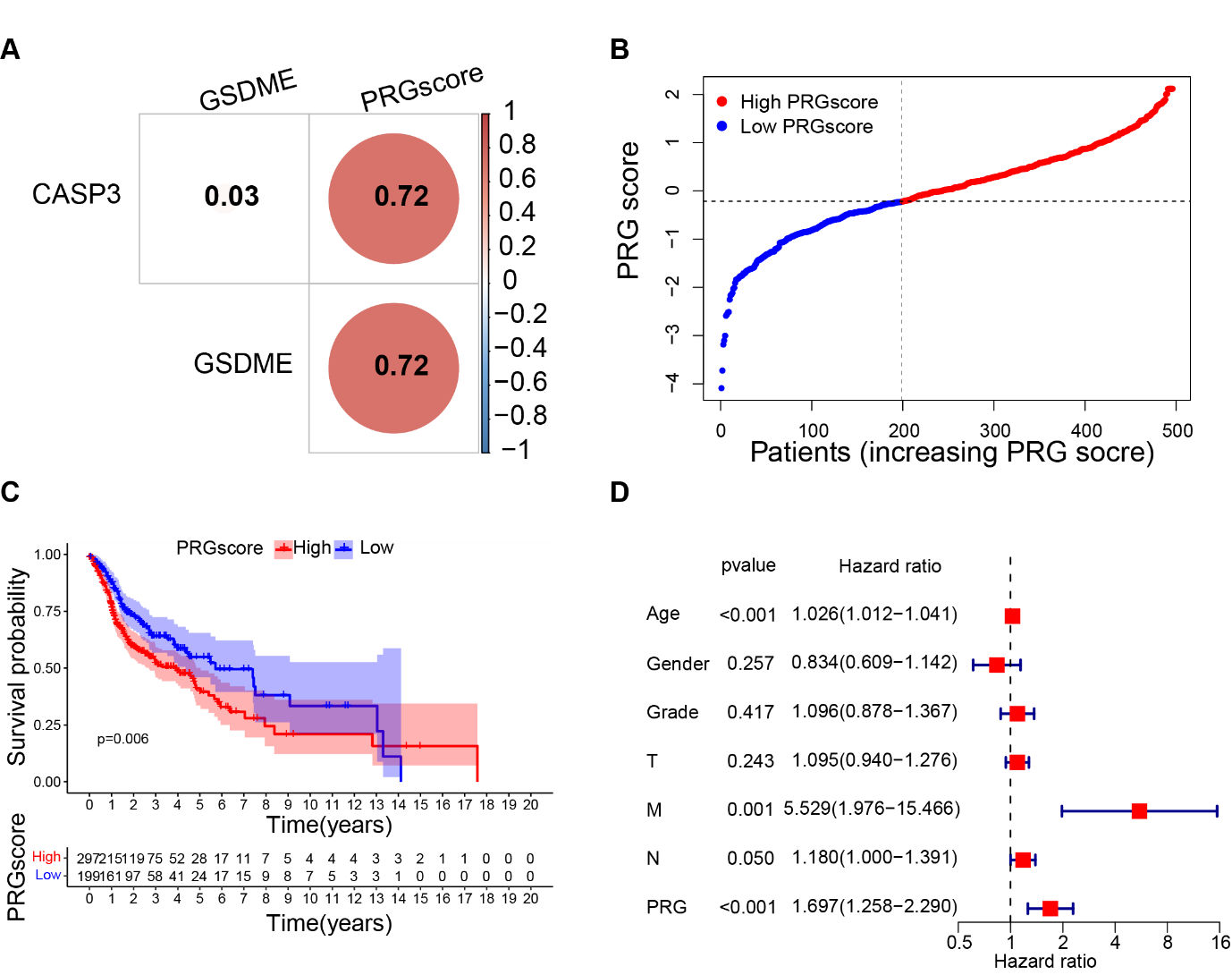
**

**Fig. S5. Construction of the pyroptosis-related prognostic signature (TCGA cohort) based on 2 genes.** (A) Pearson correlation analysis between 2 pyroptosis-related genes and PRGscore. (B) PRGscore of HNSCC samples premised on the cutpoint (the left of the dotted line signifies the low PRGscore group while the right side signifies the high PRGscore group). (C) Kaplan-Meier survival analysis of low and high PRGscore subgroups; the dashed lines represent the 95% confidence interval. (D) Multivariate Cox regression analysis of the PRGscore and clinicopathological parameters in HNSCC samples. Abbreviations: TCGA, The Cancer Genome Atlas; Caspase-3; GSDME, Gasdermin E; PRGscore, Pyroptosis-Related Gene Score; HNSCC, head and neck squamous cell carcinoma.

**Figure S6 Correlation between PRGscore and chemotherapy**

**
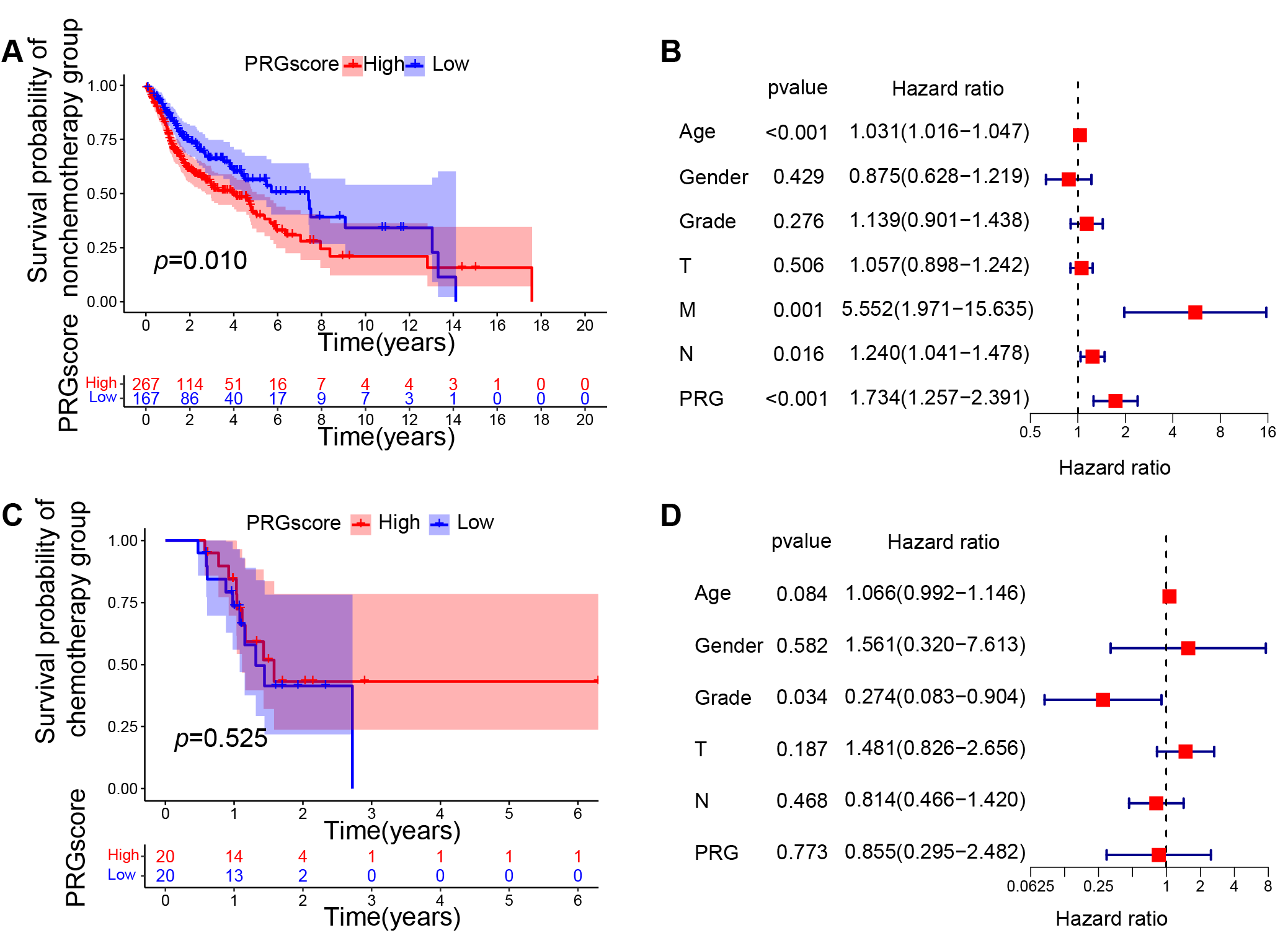
**

**Fig. S6. Correlation between PRGscore and chemotherapy.** (A) Kaplan-Meier curves of low and high PRGscore groups in HNSCC patients not receiving chemotherapy; the dashed lines represent the 95% confidence interval. (B) Multivariate Cox regression analysis of the PRGscore (as a categorical variable) in HNSCC patients not receiving chemotherapy. (C) Kaplan-Meier curves of low and high PRGscore groups in HNSCC patients adopting chemotherapy; the dashed lines represent the 95% confidence interval. (D) Multivariate Cox regression analysis of the PRGscore (as a categorical variable) in HNSCC patients adopting chemotherapy. Abbreviations: PRGscore, Pyroptosis-Related Gene Score.

**Figure S7 Correlation between PRGscore and chemotherapy sensitivity through pRRophetic R package and verified in HNSCC cell lines
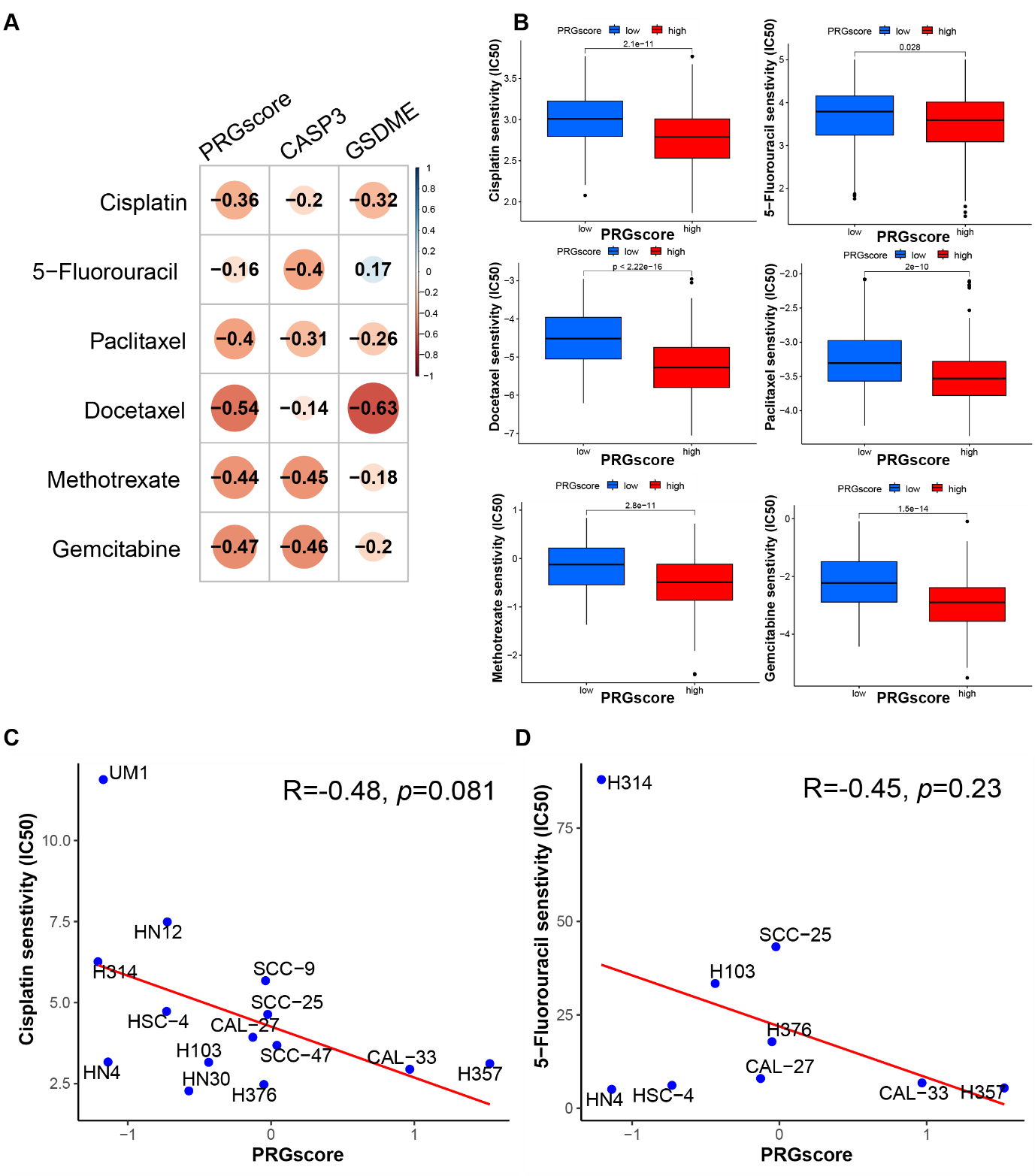
**

**Fig. S7. Correlation between PRGscore and chemotherapy sensitivity through pRRophetic R package and verified in HNSCC cell lines.** (A)Chemotherapy sensitivity prediction analysis through pRRophetic R package. (B) Drug sensitivity analysis of Chemotherapy for HNSCC patients between high- and low-PRGscore cohorts. (C)Pearson correlation analysis between the PRGscore and the IC50 of cisplatin in HNSCC cell lines. (D) Pearson correlation analysis between the PRGscore and the IC50 of 5-Fluorouracil in HNSCC cell lines. Abbreviations: Caspase-3; GSDME, Gasdermin E; PRGscore, Pyroptosis-Related Gene Score.

**Supplemental Table 1. The clinicopathological features of high PRGscore and low PRGscore group.**

| **Clinicopathological Variables** | **Overall** | **Low PRGscore group** | **High PRGscore group** | ***P*** |
| --- | --- | --- | --- | --- |
| N | 496 | 294 | 202 |  |
| PRG group (%) |  |  |  |  |
| Low PRGscore | 294 (59.3) | 294 (100.0) | 0 (0.0) |  |
| High PRGscore | 202 (40.7) | 0 (0.0) | 202 (100.0) |  |
| Age (%) |  |  |  |  |
| <=65 | 322 (64.9) | 191 (65.0) | 131 (64.9) | 1 |
| >65 | 174 (35.1) | 103 (35.0) | 71 (35.1) |  |
| Gender (%) |  |  |  |  |
| Male | 364 (73.4) | 223 (75.9) | 141 (69.8) | 0.163 |
| Female | 132 (26.6) | 71 (24.1) | 61 (30.2) |  |
| Grade (%) |  |  |  |  |
| G1 | 61 (12.9) | 43 (15.4) | 18 (9.2) | 0.106 |
| G2 | 295 (62.2) | 172 (61.6) | 123 (63.1) |  |
| G3 | 118 (24.9) | 64 (22.9) | 54 (27.7) |  |
| Stage (%) |  |  |  |  |
| Stage I | 19 (3.9) | 14 (4.9) | 5 (2.5) | 0.263 |
| Stage II | 94 (19.5) | 50 (17.5) | 44 (22.3) |  |
| Stage III | 102 (21.2) | 65 (22.8) | 37 (18.8) |  |
| Stage IV | 267 (55.4) | 156 (54.7) | 111 (56.3) |  |
| T (%) |  |  |  |  |
| T1 | 33 (6.9) | 22 (7.7) | 11 (5.6) | 0.835 |
| T2 | 143 (29.7) | 84 (29.6) | 59 (29.9) |  |
| T3 | 129 (26.8) | 75 (26.4) | 54 (27.4) |  |
| T4 | 176 (36.6) | 103 (36.3) | 73 (37.1) |  |
| M (%) |  |  |  |  |
| M0 | 466 (98.9) | 278 (99.3) | 188 (98.4) | 0.665 |
| M1 | 5 (1.1) | 2 (0.7) | 3 (1.6) |  |
| N (%) |  |  |  |  |
| N0 | 238 (50.2) | 138 (49.3) | 100 (51.5) | 0.399 |
| N1 | 80 (16.9) | 54 (19.3) | 26 (13.4) |  |
| N2 | 151 (31.9) | 85 (30.4) | 66 (34.0) |  |
| N3 | 5 (1.1) | 3 (1.1) | 2 (1.0) |  |

**Abbreviations**: PRGscore, Pyroptosis-Related Gene Score.

**Table S2. Cox proportional hazards assumption test on various cohorts of HNSCC.**

| **Variates** | | **TCGA cohort** | | **TCGA-nonchemotherapy cohort** | | | **TCGA-chemotherapy cohort** | |
| --- | --- | --- | --- | --- | --- | --- | --- | --- |
|  | chisq | | *p* | | chisq | *p* | chisq | *p* |
| Age | 0.0508 | | 0.82 | | 0.4687 | 0.49 | 0.0718 | 0.79 |
| Gender | 0.1582 | | 0.69 | | 0.0134 | 0.91 | 0.0592 | 0.81 |
| Grade | 0.7112 | | 0.4 | | 1.1108 | 0.29 | 1.0653 | 0.3 |
| T | 0.211 | | 0.65 | | 0.1802 | 0.67 | 0.111 | 0.74 |
| M | 0.465 | | 0.5 | | 0.828 | 0.36 | NA | NA |
| N | 0.7702 | | 0.38 | | 0.8934 | 0.34 | 2.4367 | 0.12 |
| PRGscore | 0.415 | | 0.52 | | 0.3075 | 0.58 | 0.038 | 0.85 |
| GLOBAL | 3.2754 | | 0.86 | | 5.3125 | 0.62 | 5.5922 | 0.47 |

**Abbreviations**: PRGscore, Pyroptosis-Related Gene Score.
